# Efficacy of dihydroartemisinin–piperaquine to prevent malaria and complication-related malaria during pregnancy compared to sulfadoxine–pyrimethamine: a systematic review, meta-analysis, and meta-regression

**DOI:** 10.1017/S0950268826101939

**Published:** 2026-07-08

**Authors:** Artha Maressa Theodora Simanjuntak, Frengki Prabowo Saputro Wijayanto, Randika Taufiq Hari Nugraha, Firman Hanif Prathama, Roy Novri Ramadhan, Taufik Mulya Perdana, Jonathan Hasian Haposan, Annisa Dewi Nugrahani, Yovita Hartantri, Sidik Maulana

**Affiliations:** 1 Department of Medicine, Faculty of Medicine Public Health and Nursing, Gadjah Mada University, Indonesia; 2 Department of Medicine, Faculty of Medicine, Airlangga University, Indonesia; 3 Department of Parasitology, Faculty of Medicine, Public Health, and Nursing, Gadjah Mada University, Indonesia; 4 Department of Biostatistics, Epidemiology, and Population Health, Faculty of Medicine, Public Health and Nursing, Gadjah Mada University, Indonesia; 5 Doctoral Program of Medicine, Faculty of Medicine, Padjadjaran University, Indonesia; 6 Department of Internal Medicine, Faculty of Medicine, Padjadjaran University, Indonesia; 7 Doctor Program of Nursing, Faculty of Nursing, Padjadjaran University, Indonesia; 8 Department of Immunology and Infectious Disease, Dr. Hasan Sadikin General Hospital , Indonesia; 9 Research Center for Care and Control of Infectious Disease (RC3ID) Universitas Padjadjaran

**Keywords:** dihydroartemisinin–piperaquine, intermittent preventive treatment of malaria in pregnancy, malaria prevention, pregnant women, SDGs-3, sulfadoxine–pyrimethamine

## Abstract

This study aimed to evaluate the efficacy of DHP as an alternative to SP for the prevention of malaria and malaria-related complications during pregnancy. A systematic review and meta-analysis were conducted using pooled effect sizes extracted from published studies. Compared to SP, DHP significantly reduced maternal malaria infection by 78% (RR 0.22, 95% CI: 0.09–0.55), placental malaria infection by 73% (RR 0.27, 95% CI: 0.08–0.98), and maternal anaemia by 16% (RR = 0.84, 95% CI: 0.79–0.89). Women randomized to the DHP arm had a 28% lower risk of foetal loss (RR 0.72, 95% CI: 0.40–1.31), a 4% lower risk of low birth weight (RR 0.96, 95% CI: 0.69–1.32), and a 17% lower risk of preterm births compared with those receiving SP (RR 0.83, 95% CI: 0.61–1.13), though the reduction was not statistically significant. Therefore, DHP appears to be more effective than SP as IPTp therapy for reducing maternal malaria infection, placental malaria infection, and maternal anaemia. However, this study does not significant advantage of DHP over SP in reducing adverse birth outcomes.

## Summary

Increasing resistance of sulfadoxine–pyrimethamine (SP) has reduced its effectiveness as intermittent preventive treatment of malaria during pregnancy (IPTp) to prevent malaria in pregnancy. Thereby, this systematic review and meta-analysis evaluated the efficacy of dihydroartemisinin–piperaquine (DHP) compared to SP for IPTp. A comprehensive search of PubMed, Scopus, CENTRAL, and Google Scholar identified seven randomized controlled trials involving a total of 8132 pregnant women. The meta-analysis suggests that DHP significantly reduced the risk of maternal malaria infection, placental malaria infection, and maternal anaemia. However, no statistically significant differences were observed between DHP and SP in the outcomes of foetal loss, low birth weight, or preterm birth. These findings suggest that DHP offers superior benefit over SP for mothers and their baby. Given the increasing resistance to SP and the ongoing need for effective IPTp regimens, DHP may represent a promising alternative, especially in high-transmission or SP-resistant areas. This study supports the potential revision of current IPTp guidelines to include DHP as a more effective preventive option during pregnancy.

## Key results and importance

- DHP significantly reduced maternal malaria infection by 78% and placental malaria infection by 73% compared to SP.
- Maternal anaemia risk was also significantly reduced with DHP, which is crucial for improving maternal health outcomes.
- No significant differences were found in foetal outcomes (e.g. foetal loss, low birth weight, preterm birth).
- DHP may be a promising alternative to SP in IPTp programmes, especially in areas with SP resistance or high malaria burden.
- The study supports re-evaluation of WHO IPTp guidelines, providing evidence-based rationale for DHP inclusion.

## Introduction

Malaria is a global health challenge that significantly affects the economy, particularly in underdeveloped areas [1]. This disease is caused by the *Plasmodium* parasite and is transmitted through the bite of *Anopheles* mosquitoes [2, 3]. According to the World Malaria Report, there are 282 million malaria cases and 610000 deaths in 2024, showing an increasing trend compared to previous years [4]. According to the World Health Organization (WHO), approximately half of the global population is at high risk of malaria infection, particularly vulnerable groups such as infants, children, pregnant women, and individuals with HIV/AIDS [5].

A previous meta-analysis of 253 studies estimated the overall prevalence of malaria during antenatal visits to be 20.09% and at delivery to be 17.32%, with the highest malaria in pregnancy (MIP) cases found in Africa [6]. A multi-centre cross-sectional study among pregnant women at first antenatal care visit in Ghana reported an anaemia prevalence of 42.4%, with 35.7%, 6.1%, and 0.6% presenting with mild, moderate, and severe anaemia, respectively [7]. Maternal anaemia and severe maternal outcomes are primarily driven by systemic parasitaemia, haemolysis, bone marrow suppression, and other complications of severe malaria [8]. In malaria-endemic areas, approximately 20–40% of the first pregnancies result in babies with low birth weight (LBW) [9]. These findings suggest the importance of effective malaria prevention and treatment strategies during pregnancy to safeguard maternal and neonatal health.

Although malaria vaccines, such as RTS, S/AS01, and R21/Matrix-M, have been approved for use in children, no licensed vaccine is currently available for adults or pregnant women [10]. Therefore, preventive chemotherapy and vector control remain mainstay strategies for malaria prevention [11]. Intermittent preventive treatment in pregnancy (IPTp) has been proven effective in reducing morbidity and mortality from malaria [12]. IPTp involves routine administration of antimalarial drugs to pregnant women at specific time intervals [13]. WHO recommends the use of sulfadoxine–pyrimethamine (SP) as IPTp for malaria, specifically *Plasmodium falciparum* [14]. SP inhibits folic acid synthesis, which is an important compound for parasite growth [15].

However, the effectiveness of IPTp-SP has declined in several endemic regions due to the emergence and spread of antifolate resistance, especially in several countries across Africa [16]. Resistance is mediated by mutations in the *dhfr* and *dhps* genes, which encode the molecular targets of pyrimethamine and sulfadoxine, respectively [17]. The combination of *dhfr* triple mutations with key *dhps* mutations forms a quintuple mutant haplotype strongly associated with SP treatment failure and approaching fixation in some areas in Africa [18]. In contrast, West Africa exhibits high *dhfr* mutation prevalence but more variable *dhps* mutation frequencies, resulting in regional differences in SP efficacy [19]. This heterogeneity in resistance patterns raises important questions regarding the continued protective benefit of IPTp-SP across diverse epidemiological settings.

Artemisinin-based combination therapy (ACT) has been extensively evaluated for the treatment of uncomplicated *P. falciparum* malaria in pregnancy [14]. ACT combines two or more drugs with different mechanisms to increase the efficacy and prevent resistance [20]. Some commonly used ACT combinations include artemether–lumefantrine, artesunate–amodiaquine, and dihydroartemisinin–piperaquine (DHP) [21]. DHP has become the focus of research for chemoprevention, not only because of its high efficacy and tolerability against *P. falciparum*, but also because of its very long elimination half-life, which provides a prolonged post-treatment prophylactic effect [22].

More recently, an individual participant data meta-analysis published in 2025 by Roh et al. [23] has further explored the efficacy and safety of DHP in IPTp. However, substantial heterogeneity across transmission intensity and resistance settings remains insufficiently explored. Therefore, the present aggregate data meta-analysis aims to update the efficacy of DHP as an alternative to SP for IPTp and to apply meta-regression analyses to investigate potential sources of heterogeneity across different malaria transmission and resistance contexts. The secondary aim of this study was to evaluate malaria-associated birth outcomes.

## Methods

### Study design

This systematic review and meta-analysis followed the Preferred Reporting Items for Systematic Review and Meta-Analysis (PRISMA) guidelines and the Cochrane Handbook for Systematic Reviews of Interventions, version 6.3, 2022 [24, 25]. This study was prospectively registered in the International Prospective Register of Systematic Reviews (PROSPERO) (number CRD420251021431).

### Study eligibility criteria

Inclusion criteria were determined based on the PICOS framework (population, intervention, comparison, outcome, and study design). This study included randomized controlled trials (RCTs) that enrolled pregnant women who were tested for malaria. The intervention focused on the administration of DHP compared to SP as IPTp. Trials with multiple intervention arms that directly compared DHP and SP as IPTp were included, regardless of the dosing frequency. However, non-IPTp regimens, such as intermittent screening and treatment (ISTp), or curative treatment-only regimens were excluded. This aggregate data meta-analysis relied exclusively on published study-level summary data. No individual participant data or unpublished outcome data were requested from study authors. However, for inaccessible full-text articles, we attempted to contact corresponding authors to obtain the published manuscript. The primary outcomes of interest were maternal and placental malaria infections, both confirmed by microscopy [26]. Studies reporting at least one of these primary outcomes were considered eligible for inclusion. The secondary outcomes included maternal anaemia and pregnancy complications, specifically foetal loss, low birth weight (LBW), and preterm birth. Studies were included if they reported any primary or secondary outcome. The exclusion criteria for this study were as follows: (1) unpublished studies, including conference abstracts without full text; (2) publications prior to 2015; (3) full-text articles that could not be accessed and the authors did not respond to two requests via email; (4) studies in languages other than English and Indonesia; (5) studies enrolling pregnant women who had received either SP or DHP during their current pregnancy at the time of recruitment; and (6) study populations infected with HIV.

### Outcome endpoints

#### Primary outcomes

The primary outcomes were maternal and placental malaria infections. Maternal malaria infection was defined as the detection of *P. falciparum* or *Plasmodium vivax* in peripheral blood during pregnancy or delivery, most commonly identified by light microscopy, with polymerase chain reaction (PCR) used for confirmation in some studies have been used for confirmation. Moreover, placental malaria was defined as the presence of parasites in placental blood identified by microscopy or PCR or in placental tissue identified by histopathological examination. However, for consistency across studies, we extracted placental malaria infections identified by microscopy, as this was the only diagnostic method uniformly reported across all included trials.

#### Secondary outcomes

The secondary outcomes included maternal anaemia and pregnancy complications such as foetal loss, LBW, and preterm birth. Maternal anaemia was defined according to WHO thresholds of haemoglobin concentration <11 g/dL in the first and third trimesters and <10.5 g/dL in the second trimester. Foetal loss was defined as miscarriage (<28 gestational weeks), spontaneous abortion, or stillbirth (≥28 gestational weeks), which were summed when all three were presented separately. LBW was defined as birth weight <2500 g. Preterm birth was defined as delivery at <37 weeks of gestation.

### Search strategy and selection process

Literature was systematically searched across three databases: PubMed, Scopus, and Cochrane Central Register of Controlled Trials (CENTRAL). In addition, we systematically searched the literature using Google Scholar. The literature search was conducted using keywords with the basic Boolean operators ‘AND’ and ‘OR’, as detailed in Supplementary File S1. All of these terms were selected in accordance with the Medical Subject Headings (MeSH), all of which were imported into Rayyan.ai, and duplicates were removed. Titles and abstracts were assessed by two independent reviewers (AMTS and FPSW), and any disagreements were resolved in consultation with a third author (RTHN). The selected articles were assessed for full text based on pre-defined eligibility criteria. Disagreements during the selection process were resolved through discussion with a third reviewer (SM).

### Data extraction and quality assessment

Data extraction from each included study is presented using a table consisting of author, year of publication, country, study design, sample size, demographic data (sample size and mean age), intervention, and control (name, dose, administration time, and follow-up time). The data were extracted independently by two reviewers (AMTS and RTHN). Furthermore, the third reviewer (FPSW) verified the accuracy of the extracted data and performed statistical analysis. The number of events of five outcomes, maternal malaria infection, placental malaria infection, foetal loss, LBW, preterm birth, and maternal anaemia in the intervention (DHP) and control (SP) groups, were also extracted.

Risk of bias was analysed using the Revised Cochrane Risk of Bias (RoB) tool for RCTs, which included five domains and was used to assess potential bias in the studies included in the analysis. Two reviewers (AMTS and FPSW) independently assessed the potential bias using a formula developed by the Cochrane Collaboration. Subsequently, the results were entered into a bias domain file (. xlsx), and then uploaded to the ROBVIS website to visualize the final results. The quality assessment was performed independently by two reviewers (AMTS and FPSW), and any disagreements were resolved through discussion, focusing on the reasons for disagreement to reach a consensus.

### Statistical analysis

Statistical analysis was conducted using RStudio (version 4.4.1, The R Foundation for Statistical Computing, Vienna, Austria) with the ‘meta’ and ‘metafor’ package. This study was performed as an aggregate data meta-analysis using study-level summary data extracted from the included trials. A random-effects Mantel–Haenszel model was used to estimate the risk ratio (RR) with a 95% confidence interval (CI) and *p* values. The *I* ^2^ statistic was used to analyse heterogeneity, with criteria thresholds of 0%, 25%, 50%, and 75% representing non-significant, low, moderate, and high heterogeneity, respectively [25]. Additionally, leave-one-out analysis was used for sensitivity analysis to ensure the robustness of the analysis. Publication bias was performed using the DOI plot because less than 10 studies were included [27]. Exploratory meta-regression analyses were conducted to investigate the potential sources of heterogeneity. Categorical variables were modelled as binary indicators, whereas continuous variables were analysed, as reported in the original studies. The following study-level covariates were examined: (1) percentage of primigravid participants, (2) mean maternal age in years at the time of enrolment, (3) publication year, categorized as <2020 and ≥2020; (4) dosing schedule, categorized as three doses and monthly; (5) resistance setting, categorized as low and high, as defined according to the primary studies; and (6) geographic region, categorized as East Africa and West Africa.

## Results

### Study selection

**Figure 1.**
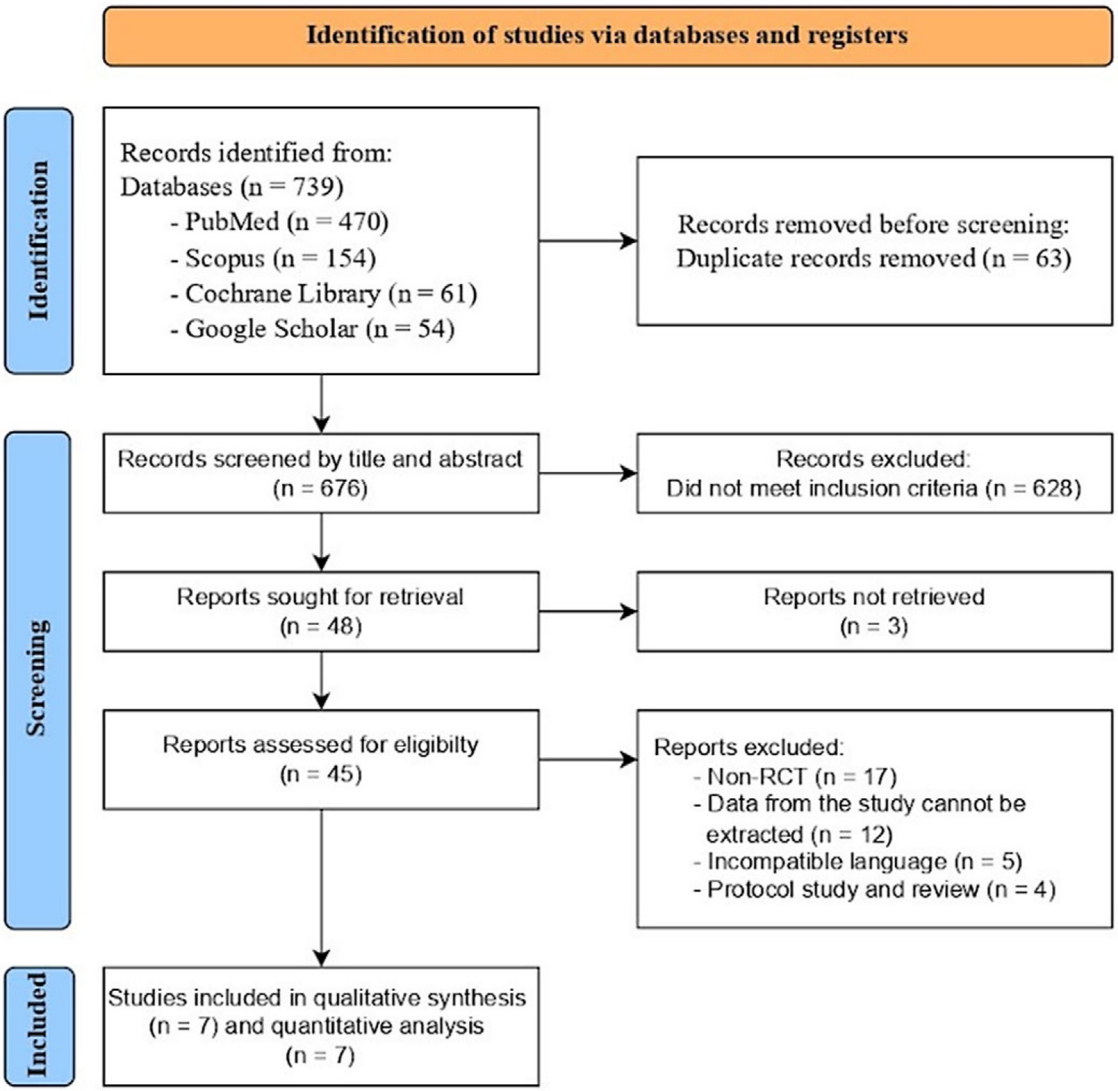
Study selection flow. Figure 1. long description.

A total of 739 studies were retrieved from three databases and one search engine. Before screening, 63 studies were excluded because they were duplicates. A total of 676 articles were excluded during the title screening, leaving 48 articles. Additionally, three articles were excluded because full-text not retrieved. Further assessments were performed on the 45 articles for full-text eligibility. Thirty-eight articles were excluded because of unsuitable study design and data extraction, incompatible language, or review articles. Ultimately, seven studies were included in the meta-analysis. A summary of the screening and selection processes by assessing eligibility criteria is presented in Figure 1.

### Demographic characteristics of included studies

A total of 8132 pregnant women were included in the analysis. This study included studies published from 2015 to 2025, conducted in several countries (i.e., including Kenya, Malawi, Nigeria, Tanzania, and Uganda) [28, [29], [30], [31], [32], [33], 34]. The included studies involved pregnant women aged at least 15 years and were randomized into intervention or control groups. The detailed demographic characteristics of the included studies are shown in Table 1.

**Table 1.** Demographic characteristics of included studies Table 1. long description.

Study	Country	Resistance level	Study design	Intervention	Control	Population	Age (years)	BMI
Desai et al. [28]	Kenya	High	RCT, open-label	DHP	SP	514 514	23.4 ± 5.5 23.5 ± 6.0	22.9 ± 3.6 22.8 ± 3.6
Kajubi et al. [29]	Uganda	High	RCT, double-blind	DHP	SP	391 391	23 ± 5.93 23 ± 5.93	NA NA
Kakuru et al. [30]	Uganda	High	RCT, double-blind	DHP	SP	94 106	22.2 ± 4.3 21.3 ± 3.6	NA NA
Kakuru et al. [31]	Uganda	High	RCT, double-blind	DHP	SP	918 920	24.7 ± 6.3 24.5 ± 6.2	NA NA
Madanitsa et al. [32]	Malawi, Tanzania, Kenya	High	RCT double-blind	DHP	SP	1561 1561	25.1 ± 6.1 24.9 ± 6.1	24.4 ± 4.8 24.1 ± 4.8
Mlugu et al. [33]	Tanzania	High	RCT, open-label	DHP	SP	478 478	27.2 ± 6.1 27.3 ± 6.2	25.8 ± 6.1 25.5 ± 6.4
Okoro et al. [34]	Nigeria	Low	RCT, double- blind	DHP	SP	112 94	27.3 ± 5.7 27.7 ± 6.1	25.2 ± 6.0 26.0 ± 6.3

*Note:* Data were presented as mean ± SD. Resistance level was determined based on molecular marker prevalence and resistance patterns described in the primary studies. BMI, body mass index; DHP, dihydroartemisinin–piperaquine; RCT, randomized controlled trial; SP, sulfadoxine–pyrimethamine; .

### Clinical characteristics of included studies

Maternal and placental malaria infections were detected microscopically according to the gold standard [26]. The drug was administered during the second and third trimesters of pregnancy. All included trials used fixed-dose regimens consistent with WHO-aligned dosing for DHP and SP. However, the IPTp dosing schedule varied between studies, with regimens administered either as three scheduled doses or monthly until delivery. Additionally, the follow-up time differed across the studies. A summary of the data extraction process is presented in Table 2.

**Table 2.** Clinical characteristics of included studies Table 2. Long description.

Study	Follow-up (weeks)	Treatment	Dose (1 tablet)	Time	Gravida status (*n*/*N*)	
					Primi	Multi
Desai et al. [28]	4	DHP	Dihydroartemisinin 40 mg and piperaquine 320 mg	Three tablets are equivalent to one dose, taken orally once daily. Treatment for 3 consecutive days.	263/514	251/514
		SP	Sulfadoxine 500 mg and pyrimethamine 25 mg	Three tablets are equivalent to one dose, taken orally once daily. Treatment for 1 day.	292/514	222/514
Kajubi et al. [29]	6	DHP	Dihydroartemisinin 40 mg and piperaquine 320 mg	Three tablets are equivalent to one dose, taken orally once daily. Treatment for 3 consecutive days.	93/391	298/391
		SP	Sulfadoxine 500 mg and pyrimethamine 25 mg	Three tablets are equivalent to one dose, taken orally once daily. Treatment for 1 day.	102/391	289/391
Kakuru et al. [30]	6	DHP	Dihydroartemisinin 40 mg and piperaquine 320 mg	Three tablets are equivalent to one dose, taken orally once daily. Treatment for 3 consecutive days.	33/94	61/94
		SP	Sulfadoxine 500 mg and pyrimethamine 25 mg	Three tablets are equivalent to one dose, taken orally once daily. Treatment for 1 day.	42/106	64/106
Kakuru et al. [31]	4	DHP	Dihydroartemisinin 40 mg and piperaquine 320 mg	Three tablets are equivalent to one dose, taken orally once a day. Treatment for 3 consecutive days	234/920	686/920
		SP	Sulfadoxine 500 mg and pyrimethamine 25 mg	Three tablets are equivalent to one dose. Taken orally once a day. Treatment for 1 day.	230/918	234/920
Madanitsa et al. [32]	4	DHP	Dihydroartemisinin 40 mg and piperaquine 320 mg	Three tablets are equivalent to one dose, taken orally once a day. Treatment for 3 consecutive days.	473/1557	1084/1557
		SP	Sulfadoxine 500 mg and pyrimethamine 25 mg	Three tablets are equivalent to one dose, taken orally once a day. Treatment for 1 day.	493/1558	1065/1558
Mlugu et al. [33]	6	DHP	Dihydroartemisinin 40 mg and piperaquine 320 mg	Three tablets are equivalent to one dose, taken orally once a day. Treatment for 3 consecutive days.	115/478	363/478
		SP	Sulfadoxine 500 mg and pyrimethamine 25 mg	Three tablets are equivalent to one dose, taken orally once a day. Treatment for 1 day.	128/478	350/478
Okoro et al. [34]	4	DHP	Dihydroartemisinin 40 mg and piperaquine 320 mg	Three tablets are equivalent to one dose, taken orally once a day. Treatment for 3 consecutive days	38/112	74/112
		SP	Sulfadoxine 500 mg and pyrimethamine 25 mg	Three tablets are equivalent to one dose, taken orally once a day. Treatment for 1 day.	33/94	61/94

*Note:* DHP, dihydroartemisinin–piperaquine; LBW, low birth weight; SP, sulfadoxine–pyrimethamine.

### Quality assessment

**Figure 2.**
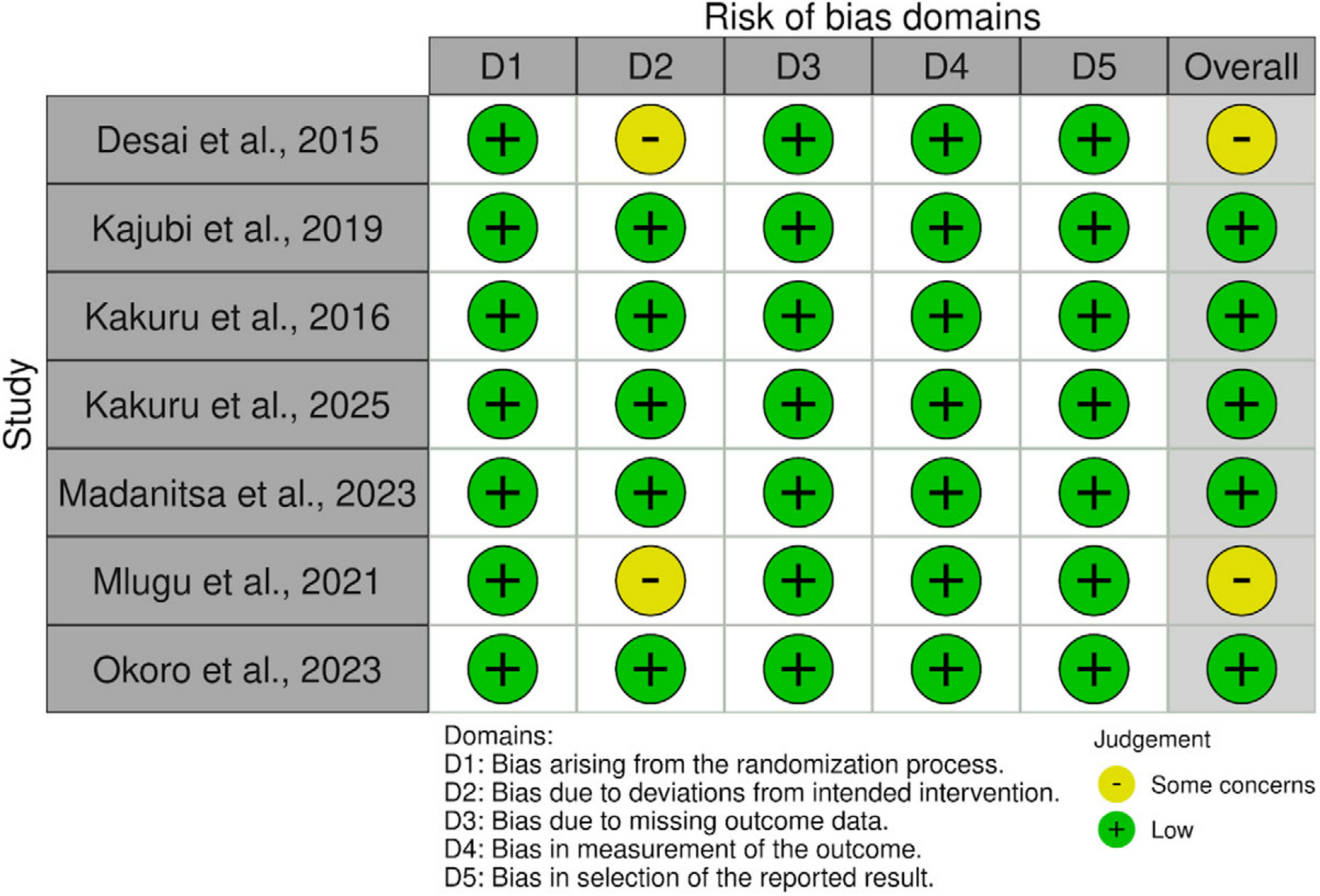
The assessment of bias risk was conducted using the revised tool for risk of bias in randomized controlled trial of intervention for seven included studies included in this study. Figure 2. long description.

The results of the risk of bias assessment are shown in Figure 2. All studies had a low risk of bias arising from the randomization process, while two had a moderate risk of bias due to deviations from the intended intervention, as these trials were conducted using an open-label design, potentially influencing participant behaviour and outcome assessment. The other five studies had a low risk of bias. All studies had a low risk of bias due to missing outcome data, outcome measurements, and selection of the reported results. Overall, approximately 72% of the included studies were judged to have low risk of bias and 28% to have moderate risk of bias.

### Efficacy of DHP in comparison to SP to prevent malaria infection

**Figure 3.**
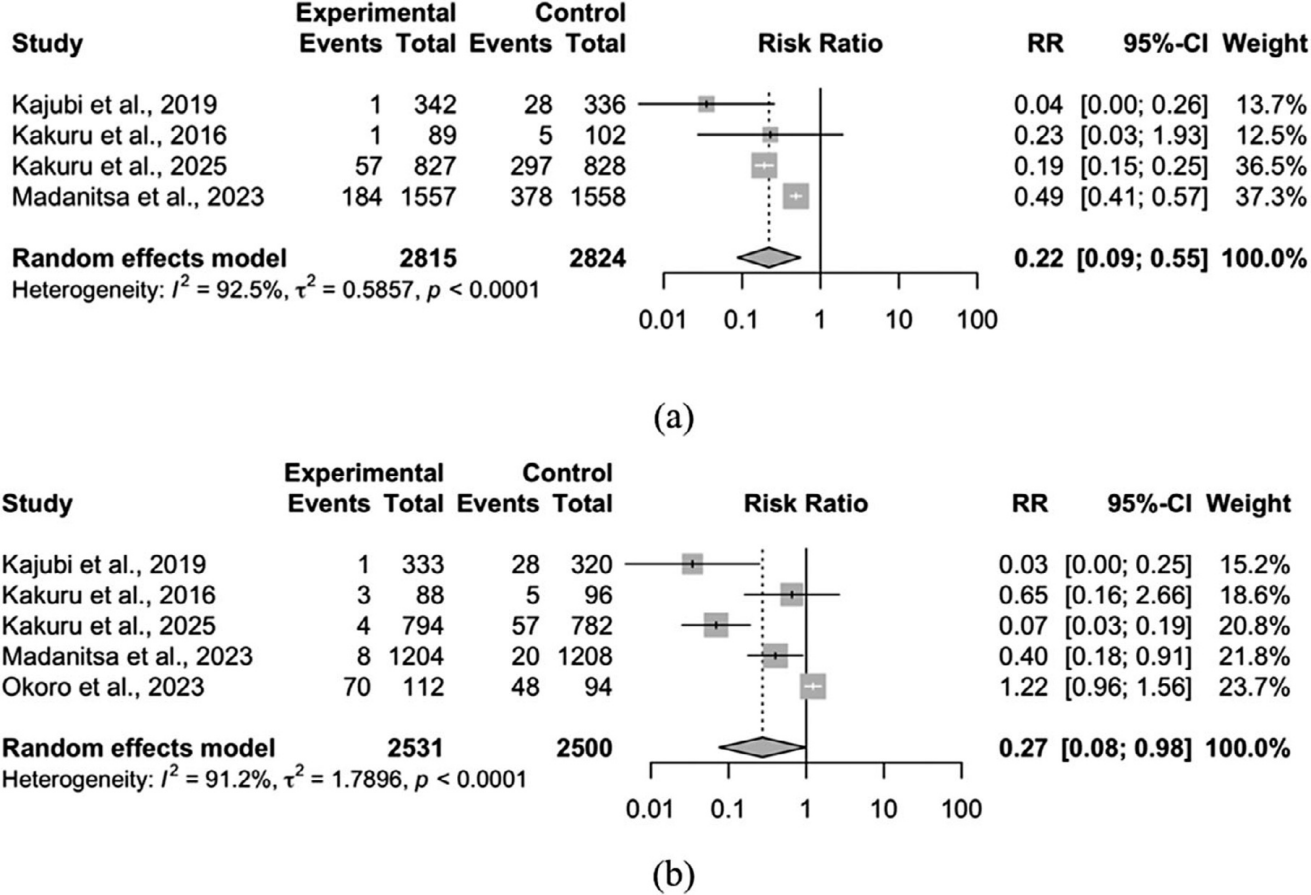
Efficacy of dihydroartemisinin–piperaquine compared to sulfadoxine–pyrimethamine in maternal malaria infection (a) and placental malaria infection (b). Figure 3. long description.

The effectiveness of DHP compared with SP in reducing maternal and placental malaria infections was reported in four studies, as shown in Figure 3. The meta-analysis showed that DHP significantly reduced the incidence of maternal malaria infection by 78% compared with SP (RR 0.22, 95% CI: 0.09–0.55; *p =* 0.0013), with substantial heterogeneity (*I* ^2^ = 92.5%). A similar trend was observed for placental malaria infection, with DHP showing a 73% reduced risk compared to SP (RR 0.27, 95% CI: 0.08–0.98; *p* = 0.0473), with substantial heterogeneity (*I* ^2^ = 91.2%).

### Efficacy of DHP in comparison to SP to prevent pregnancy complications

**Figure 4.**
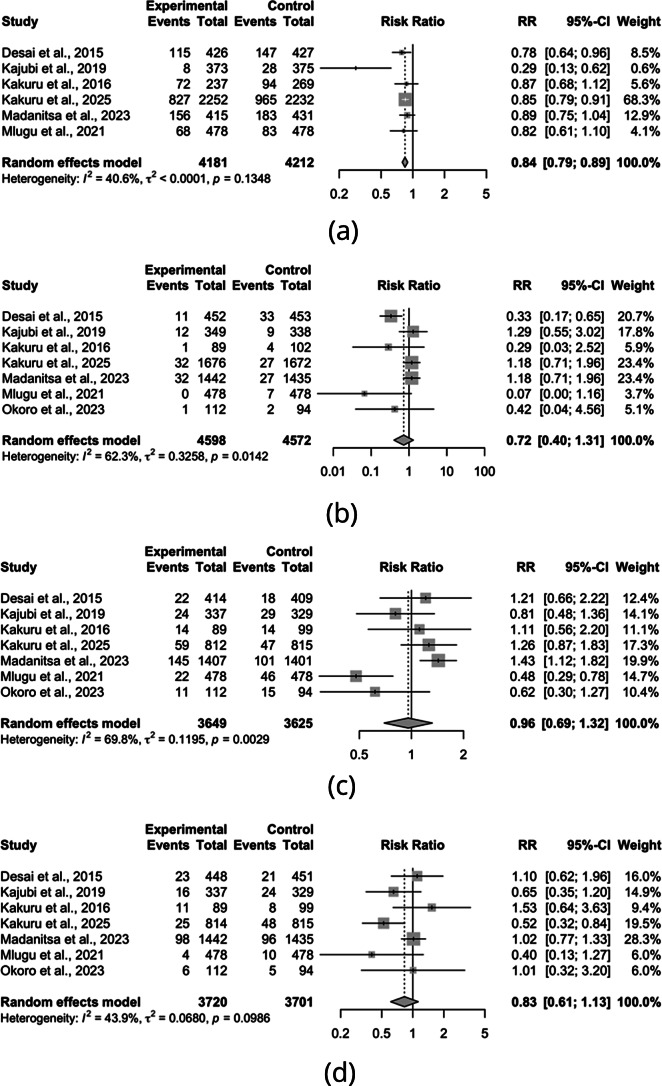
Efficacy of dihydroartemisinin–piperaquine compared to sulfadoxine–pyrimethamine on maternal anaemia (a), foetal loss (b), low birth weight (c), and preterm (d). Figure 4 long description.

The efficacy of DHP compared with SP in reducing pregnancy complications is shown in Figure 4. Compared with SP, DHP significantly reduced the risk of maternal anaemia by 16% (RR 0.84, 95% CI: 0.79–0.89, *p <* 0.0001; *I* ^2^ = 40.6%). Although not statistically significant, DHP reduced the incidence of foetal loss by 28% (RR 0.72, 95% CI: 0.40–1.31; *p* = 0.2861; *I* ^2^ = 62.3%), incidence of LBW by 4% (RR 0.96, 95% CI: 0.69–1.32; *p* = 0.7936; *I* ^2^ = 69.8%), and preterm birth by 17% (RR 0.83, 95% CI: 0.61–1.13; *p* = 0.2384; *I* ^2^ = 43.9%) compared with SP.

### Meta-regression

Meta-regression analysis was performed to explore how the effect estimates varied according to the different covariates, as summarized in Table 3. For maternal malaria infection, none of the examined covariates significantly modified the relative treatment effect of DHP compared with SP, including mean maternal age (*p* = 0.7004), proportion of primigravid women (*p* = 0.2987), monthly administration compared with the three-dose regimen (*p* = 0.9343), and publication year ≥2020 compared with <2020 (*p* = 0.2000). However, this meta-regression is not statistically uninterpretable due to limited number of studies and substantial residual heterogeneity remained.

**Table 3.** Meta-regression of covariates on maternal and placental malaria Table 3. long description.

Variable	Number of study	Estimate	95% CI (Lower; Upper)	*p*	*I* ^2^
**Maternal malaria**					
Mean of age (year)	3	0.1532	−0.6272; 0.9337	0.7004	95.06%
Primigravid percentage	4	11.8791	−10.5227; 34.2808	0.2987	60.65%
Administration three doses Administration monthly	1 3	−1.4731 −0.1302	−4.3246; 1.3784 −3.2289; 2.9684	Ref. 0.9343	Ref. 97.42%
Year of study <2020 Year of study ≥2020	2 2	−2.4569 1.2785	−4.1764; −0.7373 −0.6769; 3.2338	Ref. 0.2000	Ref. 94.61%
**Placental malaria**					
Mean of age (year)	4	0.1294	−0.5367; 0.7954	0.7034	89.89%
Primigravid percentage	5	30.9961	19.7553; 42.2370	<0.0001*	16.50%
Administration 3 doses Administration monthly	2 3	−0.0339 −2.0625	−1.3832; 1.3153 −3.8721; −0.2530	Ref. 0.0255*	Ref. 70.67%
High resistance area Low resistance area	4 1	−1.7426 1.9447	−3.0271; −0.4580 −0.6287; 4.5181	Ref. 0.1386	Ref. 77.91%
East Africa West Africa	4 1	−1.7426 1.9447	−3.0271; −0.4580 −0.6287; 4.5181	Ref. 0.1386	Ref. 77.91%
Year of study <2020 Year of study ≥2020	2 3	−1.7750 0.6948	−4.2092; 0.6592 −2.3226; 3.7121	Ref. 0.6518	Ref. 93.51%

* Statistically significant *p* < 0.05.

For placental malaria infection, the proportion of primigravid women was significantly associated with variation in treatment effects (*p* < 0.0001). This suggests that differences in gravidity distribution across studies may partially account for between-study heterogeneity. In addition, the coefficient for monthly administration (coded as 1) indicated that the relative treatment effect of DHP versus SP differed significantly compared with the three-dose regimen (*p* = 0.0255), consistent with potential effect modification by dosing schedule treatment. In contrast, mean maternal age (*p* = 0.7034), resistance setting (low vs. high; *p* = 0.1386), geographic region (West Africa vs. East Africa; *p* = 0.1386), and publication year (≥2020 vs. <2020; *p* = 0.6518) were not significantly associated with differences in treatment effects. Nevertheless, these findings should be interpreted with caution, as the number of studies included in each meta-regression was small, which increased the likelihood of overfitting and limited the robustness of the observed associations.

### Publication bias

**Figure 5.**
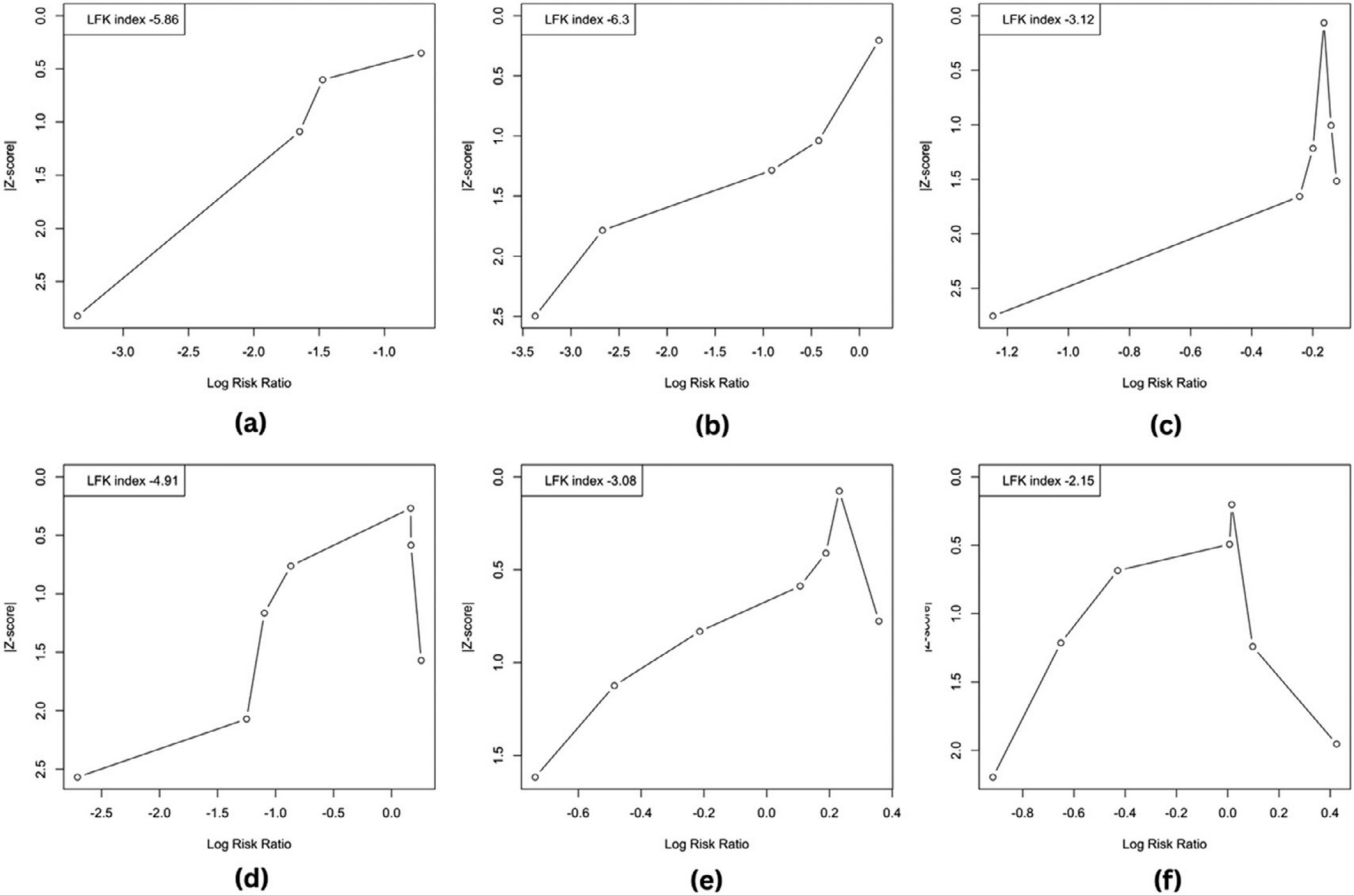
DOI plots for publication bias of maternal malaria infection (a), placental malaria infection (b), maternal anaemia (c), foetal loss (d), low birth weight (e), and preterm (f). Figure 5. long description.

The DOI plots for all the outcomes are shown in Figure 5. The DOI plot for all outcomes revealed major asymmetry, with LFK indices of −5.86 (maternal malaria infection), −6.3 (placental malaria infection), −3.12 (maternal anaemia), −4.91 (foetal loss), −3.08 (LBW), and −2.15 (preterm), indicating the potential for publication bias.

### Sensitivity analysis

The forest plots after sensitivity analysis for all outcomes are presented in Supplementary File S2. In maternal malaria infection, omitting Madanitsa et al. [32] substantially reduced heterogeneity from 92.5% to 28.4%, accompanied by an 84% lower risk compared with SP (RR = 0.16, 95% CI: 0.07–0.34; *p* < 0.0001). However, the results remain statistically significant, indicating the robustness of the findings. In placental malaria infection, though the pooled effect size remained statistically significant (RR = 0.18, 95% CI: 0.05–0.63; *p* = 0.0078), leave-one-out analysis showed that exclusion of Okoro et al. [34] reduced heterogeneity from 91.2% to 76.6%, with an 82% decrease in the pooled effect size compared with SP. Although substantial heterogeneity persisted, the robustness of the findings reduced. For foetal loss, omitting Desai et al. [28] reduced heterogeneity from 62.3% to 17.7%, with the pooled effect size remaining statistically non-significant despite a 10% increase compared with SP (RR = 1.10, 95% CI: 0.80–1.51; *p* = 0.5719). For low birth weight, exclusion of Mlugu et al. [33] reduced heterogeneity from 69.8% to 33.7%, but the pooled effect size remained statistically non-significant despite a 14% increase in effect size compared with SP (RR = 1.14, 95% CI: 0.89–1.44; *p* = 0.2965).

## Discussion

### Principal finding

This study aimed to compare the efficacy of DHP and SP for IPTp with a particular focus on maternal and foetal outcomes. Based on these findings, DHP was associated with a 78% lower risk of maternal malaria infection, a 73% lower risk of placental malaria infection, and a 16% lower risk of maternal anaemia compared with SP. Although there were no statistically significant differences, the foetal loss, LBW, and preterm birth incidences were lower in the DHP group than in the SP group. In addition, the effect size differed across covariates. For maternal malaria infection, none of the examined study-level covariates were statistically significant, while for placental malaria infection, the proportion of primigravid women and dosing schedule were significantly associated with variation in effect estimates, whereas publication year, geographic region, and resistance setting were not.

Our findings are consistent with those reported by Roh et al. [23], who confirmed the superior antimalarial efficacy of DHP compared with SP. In our aggregate data meta-analysis, DHP reduced the risk of maternal malaria infection by 78% and placental malaria by 73%, whereas Roh et al. reported a 69% reduction in clinical malaria and a 62% lower risk of placental parasitaemia. Similarly, both analyses demonstrated a statistically significant reduction in maternal anaemia, with a 16% reduction observed in our study and a 17% reduction reported by Roh et al. [23]. In contrast, the effects of DHP on reducing foetal loss, low birth weight, and preterm birth were not statistically significant in either analysis. In addition, Roh et al. [23]. observed that SP was associated with a higher mean birth weight (+50 g) and a lower risk of small-for-gestational-age infants (15% reduction), particularly among multigravidae, suggesting potential non-malarial benefits of SP. However, neither analysis demonstrated statistically significant improvements in foetal loss, low birth weight, or preterm birth. While DHP shows clear superiority in preventing malaria infection during pregnancy, current evidence does not conclusively demonstrate broad benefits for infant birth outcomes. Differences between our findings and those of Roh et al. [23] may due to varied methodological approaches, as their study used individual participant data with stratified analyses, whereas our analysis relied on aggregate data across diverse endemic settings. Moreover, our study incorporated meta-regression and sensitivity analyses, which allowed further exploration of the roles of many factors that may explain differences in interpretation. These methods allowed us to examine how associated factors, such as dosage regimen, maternal gravidity, and the level of SP resistance may influence the effectiveness of DHP compared with SP.

The reduced effectiveness of SP can largely be attributed to the growing resistance of *P. falciparum* to antifolate drugs. Mutations in the *dhfr* and *dhps* genes, which are targets of pyrimethamine and sulfadoxine, are now highly prevalent in many endemic regions [19]. Resistance is particularly pronounced in Southeast Asia and sub-Saharan Africa [35], though its distribution is heterogeneous across Africa. In East and Southern Africa, the *dhfr* triple mutant combined with key *dhps* mutations, notably K540E, has reached near fixation and is strongly predictive of SP treatment failure [19, 36]. In contrast, West Africa shows high prevalence of *dhfr* mutations but more variable *dhps* K540E frequencies, contributing to regional differences in SP efficacy [37]. These resistance patterns likely explain the diminished prophylactic benefit of SP observed in high-resistance settings.

Although variation in SP resistance is biologically plausible, our meta-regression did not show significant effect modification by resistance setting for both maternal and placental malaria. Previous studies have reported that DHP remains highly effective even in settings with multidrug-resistant *P. falciparum* [38]. Studies have shown that the combination of dihydroartemisinin and piperaquine not only provides rapid parasite clearance, but also offers extended post-treatment prophylaxis due to the long half-life of piperaquine [39]. This feature is particularly advantageous in high-transmission settings as it extends the duration of protection and reduces the frequency of malaria episodes during pregnancy.

The impact of DHP on maternal anaemia was also notable. Anaemia during pregnancy is a significant public health concern because it is associated with increased risks of maternal morbidity, foetal growth restriction, and adverse birth outcomes [40]. Our findings showed that DHP significantly reduced the prevalence of anaemia at delivery compared to SP. This effect is likely mediated by better control of parasitaemia and reduced placental sequestration of infected erythrocytes, though the precise immunological and haematological mechanisms may require further investigation [41]. The reduced incidence of parasitaemia in the DHP group is clinically important because parasitaemia, even when asymptomatic, is strongly associated with adverse maternal and neonatal outcomes [42]. Malarial parasites in the placenta can disrupt nutrient and oxygen exchange with the foetus, contributing to intrauterine growth restriction [43]. However, in terms of foetal outcomes, particularly foetal loss, LBW, and preterm birth, most trials did not observe statistically significant differences between the two drugs.

Although DHP demonstrated superior efficacy in reducing maternal and placental malaria infections, this benefit was not associated with significant improvements in neonatal outcomes, particularly foetal loss, low birth weight, or preterm delivery. Several factors could explain this discrepancy. First, much of the foetal growth impairment associated with malaria occurs early in gestation, before the initiation of IPTp, which typically begins in the second trimester [44]. Placental inflammation or vascular damage sustained early in pregnancy may only be partially reversible, even after effective parasite clearance in later trimesters. Second, other non-malarial causes of LBW and preterm birth, such as maternal undernutrition, low maternal BMI, micronutrient deficiencies, and anaemia, may attenuate the observable effect of malaria prevention on these endpoints [45].

Safety concerns related to DHP warrant careful consideration, particularly regarding its potential to prolong QTc interval. In the Ugandan trial by Kajubi et al. [29], DHP was associated with a statistically significant increase in QTc prolongation compared to SP. However, this prolongation was mild and was not linked to arrhythmias or clinically significant cardiac events. Kakuru et al. reported a higher proportion of congenital anomalies among women receiving three-dose DHP compared to SP, whereas the monthly DHP regimen showed a lower proportion [30]. Similarly, Kajubi et al. [29] found a significantly higher incidence of congenital anomalies in the DHP group than in the SP group. However, Kakuru et al. [30] emphasized that polydactyly is relatively common in African populations and is unlikely to be causally related to DHP exposure, as treatment was initiated after organogenesis and genetic factors have been postulated to explain its high frequency [31].

### Limitations

This study has several limitations that should be considered when interpreting the findings. First, the number of included trials was relatively small, which may limit the statistical power and precision of the pooled estimates, especially for secondary outcomes such as low birth weight and preterm delivery. Moreover, some sub-group and meta-regression analyses were based on a small number of studies, with certain categories represented by only a single study. This limited variability reduces the robustness and interpretability of these findings. All included studies were conducted in Africa, a region characterized by high malaria transmission and high SP resistance. This makes it difficult to determine the effectiveness of DHP in areas with lower SP resistance, thereby limiting the generalizability of our findings to other malaria endemic regions. Moreover, we extracted microscopy-based outcomes to ensure consistency across studies, as this was the only diagnostic method reported in all included trials. However, histopathology is generally more sensitive and can detect both active and past infections [46]. Therefore, relying on microscopy may have led to an underestimation of the true burden of placental malaria, particularly for sub-microscopic or previously resolved infections. In addition, adverse events were only reported qualitatively in this study because of inconsistent reporting across trials, which limited our ability to perform comprehensive pooled safety analysis. Future studies should include standardized definitions, grading, and quantitative reporting of adverse events, particularly gastrointestinal symptoms, QTc prolongation, and congenital anomalies, to strengthen the evidence on the safety of DHP in pregnancy. Furthermore, a potential risk of publication bias was observed, which could reflect the limited number of available studies, selective reporting of significant outcomes, or under-representation of smaller negative trials. An additional limitation is that this meta-analysis was based on aggregated study-level data rather than IPD, which precluded gravidity-stratified sub-group analyses. This is particularly important in malaria during pregnancy due to parity-dependent immunity. Although the proportion of primigravid participants was explored in meta-regression, study-level analyses are subject to ecological bias and cannot fully assess individual-level effect modification. Future IPD meta-analyses are needed to clarify gravidity-specific effects.

### Study implication

Cost-effectiveness is another crucial consideration in public health implementation. Although SP remains significantly cheaper than DHP, the lower upfront cost of SP must be weighed against the broader economic burden of malaria-related complications. Evidence indicates that IPTp-DP is markedly more cost-effective than IPTp-SP in regions with intense malaria transmission and high SP resistance [47]. However, these estimates were based on a smaller subset of trials and primarily focused on maternal outcomes without fully incorporating neonatal endpoints or a broader range of studies now available. With the inclusion of additional evidence in the previous meta-analysis, which found no significant differences in adverse birth outcomes between DHP and SP, the overall cost-effectiveness advantage of DHP may be less pronounced than previously estimated [47]. Nonetheless, improved maternal outcomes, particularly the reduction in malaria infection and anaemia, may still confer important indirect economic benefits through reduced healthcare utilization, improved productivity, and decreased maternal morbidity [47]. Therefore, future cost-effectiveness evaluations should integrate both maternal and neonatal outcomes, consider varying levels of SP resistance, and account for real-world implementation factors such as drug availability, adherence, and health system capacity. Such analyses are essential to accurately determine the economic feasibility and long-term public health value of replacing SP with DHP in different endemic settings.

While DHP presents a compelling alternative for IPTp, policymakers and public health officials may support consideration of DHP as an alternative IPTp regimen, particularly in settings with substantial SP resistance. The collective body of evidence, including this study, supports the potential role of DHP as an alternative to SP for IPTp. The superior efficacy of DHP in reducing risk of malaria infection and anaemia, coupled with its acceptable safety profile, make it a more effective alternative in regions with established SP resistance. While costs remain a challenge, the potential health benefits for mothers suggest that DHP may be considered as an alternative IPTp strategy, particularly in high-burden and resource-limited settings.

## Conclusion

DHP appears to be more effective than SP in reducing the risk of maternal malaria infection, placental malaria infection, and maternal anaemia during pregnancy. Although not statistically significant, the risks of LBW, foetal loss, and preterm birth were lower in the DHP group than in the SP group. Owing to the widespread resistance to sulfadoxine–pyrimethamine in malaria-endemic areas, DHP has the potential to become a new alternative IPTp regimen. Further research should prioritize areas with low-to-moderate SP resistance to confirm these findings.

## Supporting information

10.1017/S0950268826101939.sm001 Simanjuntak et al. supplementary material 1 Simanjuntak et al. supplementary material

10.1017/S0950268826101939.sm002 Simanjuntak et al. supplementary material 2 Simanjuntak et al. supplementary material

## Data Availability

No new data were generated in this review; data sharing not applicable. Full search strategies for all databases are available as supplementary material to this article.

## Supplementary material

The supplementary material for this article can be found at http://doi.org/10.1017/S0950268826101939.

## Long descriptions

### Figure 1. Long description

PRISMA flow diagram illustrating the literature search and study selection process. A total of 739 records were identified from PubMed, Scopus, CENTRAL, and Google Scholar. After removal of 63 duplicates, 676 records were excluded during title and abstract screening. Forty-eight full-text articles were assessed for eligibility. Three reports were excluded because of incomplete reporting, and 38 studies were excluded because they did not meet the eligibility criteria, including inappropriate study design, incompatible language, review articles, or insufficient data. Seven randomized controlled trials were ultimately included in the systematic review and meta-analysis.

Navigate back to Figure 1..

### Table 1. Long description

Table 1 summarizes the demographic characteristics of the seven randomized controlled trials conducted in Kenya, Uganda, Malawi, Tanzania, and Nigeria. For each study, the table reports the resistance setting, study design, intervention and control groups, sample size, mean maternal age, and body mass index. Most studies were conducted in areas with high sulfadoxine–pyrimethamine resistance. Sample sizes ranged from approximately 200 to more than 3100 participants. Mean maternal age ranged from approximately 22 to 27 years, while body mass index, where reported, was generally between 22 and 26 kg/m2.

Navigate back to Table 1..

### Figure 2. Long description

Traffic-light plot summarizing the risk of bias assessment across seven randomized controlled trials using the revised Cochrane Risk of Bias 2 tool. All studies showed low risk of bias for randomization, missing outcome data, outcome measurement, and selection of reported results. Two open-label trials showed some concerns regarding deviations from intended interventions, while the remaining studies had low overall risk of bias. Overall, five studies were judged as low risk of bias and two as having some concerns.

Navigate back to Figure 2..

### Figure 3. Long description

The image contains two panels, a and b, each showing a forest plot with columns for Study, Experimental Events and Total, Control Events and Total, Risk Ratio plot, R R, 95% C I, and Weight.

Panel a: Maternal malaria infection.

* Kajubi et al., 2019: 1/342 experimental, 28/336 control. R R 0.04 [0.00, 0.26], 13.7% weight.

* Kakuru et al., 2016: 1/89 experimental, 5/102 control. R R 0.23 [0.03, 1.93], 12.5% weight.

* Kakuru et al., 2025: 57/827 experimental, 297/828 control. R R 0.19 [0.15, 0.25], 36.5% weight.

* Madanitsa et al., 2023: 184/1557 experimental, 378/1558 control. R R 0.49 [0.41, 0.57], 37.3% weight.

* Random effects model: Total 2815 experimental, 2824 control. Pooled R R 0.22 [0.09, 0.55], 100.0% weight. Heterogeneity: I super 2 = 92.5%, tau super 2 = 0.5857, p < 0.0001.

Panel b: Placental malaria infection.

* Kajubi et al., 2019: 1/333 experimental, 28/320 control. R R 0.03 [0.00, 0.25], 15.2% weight.

* Kakuru et al., 2016: 3/88 experimental, 5/96 control. R R 0.65 [0.16, 2.66], 18.6% weight.

* Kakuru et al., 2025: 4/794 experimental, 57/782 control. R R 0.07 [0.03, 0.19], 20.8% weight.

* Madanitsa et al., 2023: 8/1204 experimental, 20/1208 control. R R 0.40 [0.18, 0.91], 21.8% weight.

* Okoro et al., 2023: 70/112 experimental, 48/94 control. R R 1.22 [0.96, 1.56], 23.7% weight.

* Random effects model: Total 2531 experimental, 2500 control. Pooled R R 0.27 [0.08, 0.98], 100.0% weight. Heterogeneity: I super 2 = 91.2%, tau super 2 = 1.7896, p < 0.0001.

The x-axis for both plots is a logarithmic scale for Risk Ratio ranging from 0.01 to 100, with a vertical line of no effect at 1.

Navigate back to Figure 3..

### Figure 4. Long description

A multi-panel figure containing four forest plots labeled a through d. Each plot includes columns for Study, Experimental Events and Total, Control Events and Total, a Risk Ratio forest plot, R R, 95 percent C I, and Weight.

* Panel a, Maternal Anaemia: Includes six studies. The random effects model shows a pooled R R of 0.84 with a 95 percent C I of 0.79 to 0.89. Heterogeneity I-squared is 40.6 percent.

* Panel b, Foetal Loss: Includes seven studies. The random effects model shows a pooled R R of 0.72 with a 95 percent C I of 0.40 to 1.31. Heterogeneity I-squared is 62.3 percent.

* Panel c, Low Birth Weight: Includes seven studies. The random effects model shows a pooled R R of 0.96 with a 95 percent C I of 0.69 to 1.32. Heterogeneity I-squared is 69.8 percent.

* Panel d, Preterm: Includes seven studies. The random effects model shows a pooled R R of 0.83 with a 95 percent C I of 0.61 to 1.13. Heterogeneity I-squared is 43.9 percent.

In all plots, a vertical solid line represents the null effect at 1.0, and a dashed vertical line indicates the pooled effect size. Individual study effects are shown as gray squares with horizontal error bars, and the aggregate effect is shown as a diamond at the bottom of each plot.

Navigate back to Figure 4..

### Table 3. Long description

Table 3 presents the meta-regression analyses exploring potential sources of heterogeneity in maternal and placental malaria outcomes. Variables examined include maternal age, proportion of primigravid women, dosing schedule, publication year, resistance setting, and geographic region. None of the evaluated variables significantly influenced the treatment effect for maternal malaria infection. For placental malaria infection, the proportion of primigravid women and the monthly dosing schedule were significantly associated with differences in treatment effect, whereas maternal age, publication year, resistance setting, and geographic region were not statistically significant.

Navigate back to Table 3..

### Figure 5. Long description

A multi-panel figure containing six line graphs arranged in two rows of three. Each graph plots Log Risk Ratio on the x-axis and the absolute value of the Z-score on the y-axis, where the y-axis is inverted with 0.0 at the top and 2.5 at the bottom.

* Panel a: Maternal malaria infection. L F K index is -5.86. The data shows a positive linear trend from a Log Risk Ratio of -3.3 at Z-score 2.8 up to -0.7 at Z-score 0.4.

* Panel b: Placental malaria infection. L F K index is -6.3. The trend shows a steady increase from a Log Risk Ratio of -3.4 at Z-score 2.5 to 0.2 at Z-score 0.2.

* Panel c: Maternal anaemia. L F K index is -3.12. The data remains at a low Z-score until a sharp peak at a Log Risk Ratio of -0.15 where the Z-score reaches 0.1.

* Panel d: Foetal loss. L F K index is -4.91. The trend shows a gradual rise followed by a sharp vertical drop at a Log Risk Ratio of approximately 0.2.

* Panel e: Low birth weight. L F K index is -3.08. The data forms an asymmetrical mountain shape, peaking at a Log Risk Ratio of 0.25 with a Z-score of 0.1.

* Panel f: Preterm. L F K index is -2.15. The data forms a broad arch, peaking near a Log Risk Ratio of 0.0 with a Z-score of 0.2, then descending as the ratio increases to 0.4.

Navigate back to Figure 5..

### Table 2. Long description

Table 2 summarizes the clinical characteristics of the included studies. For each trial, the follow-up duration, treatment regimen, drug dosage, administration schedule, and distribution of primigravid and multigravid participants are reported. Dihydroartemisinin-piperaquine was administered as three tablets once daily for three consecutive days, whereas sulfadoxine-pyrimethamine was administered as three tablets on a single day. Follow-up ranged from four to six weeks. The proportion of primigravid women varied across studies but was generally lower than that of multigravid women.

Navigate back to Table 2..
