# Supplementary material for: Efficacy of dihydroartemisinin–piperaquine to prevent malaria and complication-related malaria during pregnancy compared to sulfadoxine–pyrimethamine: a systematic review, meta-analysis, and meta-regression
Source: Epidemiol Infect. 2026 Jul 8;154:e110. doi: 10.1017/S0950268826101939 (PMC13459009; doi:10.1017/S0950268826101939)
Supplement: Supplementary file 1 [file S0950268826101939sup001.docx]

**Supplementary File 1.** Search strategy and included studies

| **Database** | **Keywords combination** | **Hits** | **Date** |
| --- | --- | --- | --- |
| PubMed | Allintitle: [(“Malaria Pregnancy” OR “Women Malaria Pregnancy”) AND (“Intermittent Preventive Treatment in Pregnancy” OR IPTp OR “Sulfadoxine-pyrimethamine” OR SP) AND  ("Dihydroartemisinin-Piperaquine" OR DP) | 470 | November 18, 2025 |
| Scopus | TITLE [(“Malaria Pregnancy” OR “Women Malaria Pregnancy”) AND (“Intermittent Preventive Treatment in Pregnancy” OR IPTp OR “Sulfadoxine-pyrimethamine” OR SP) AND  ("Dihydroartemisinin-Piperaquine" OR DP) | 154 | November 18, 2025 |
| CENTRAL | (“Malaria Pregnancy” OR “Women Malaria Pregnancy”) AND (“Intermittent Preventive Treatment in Pregnancy” OR IPTp OR “Sulfadoxine-pyrimethamine” OR SP) AND  ("Dihydroartemisinin-Piperaquine" OR DP) | 61 | November 18, 2025 |
| Google Scholar | (“Malaria Pregnancy” OR “Women Malaria Pregnancy”) AND (“Intermittent Preventive Treatment in Pregnancy” OR IPTp OR “Sulfadoxine-pyrimethamine” OR SP) AND  ("Dihydroartemisinin-Piperaquine" OR DP) | 54 | November 18, 2025 |

*Searched by Artha Maressa Theodora Simanjuntak and Frengki Prabowo Saputro Wijayanto (Gadjah Mada University, Indonesia)*
