# Supplementary material for: Efficacy of dihydroartemisinin–piperaquine to prevent malaria and complication-related malaria during pregnancy compared to sulfadoxine–pyrimethamine: a systematic review, meta-analysis, and meta-regression
Source: Epidemiol Infect. 2026 Jul 8;154:e110. doi: 10.1017/S0950268826101939 (PMC13459009; doi:10.1017/S0950268826101939)
Supplement: Supplementary file 2 [file S0950268826101939sup002.docx]

**Supplementary File 2.** Sensitivity analysis of efficacy dihydroartemisinin-piperaquine compared to sulfadoxine-pyrimethamine

1. Sensitivity analysis for maternal malaria infection

2. Sensitivity analysis for placental malaria infection

3. Sensitivity analysis for fetal loss

4. Sensitivity analysis for low birth weight
